# Challenges in management of unusual acquired factor V deficiency: A case report

**DOI:** 10.1097/MD.0000000000015259

**Published:** 2019-04-26

**Authors:** Kiyoshi Takemoto, Osamu Hamada, Koichi Kitamura, Naoki Fujiwara, Yoshitaka Miyakawa

**Affiliations:** a Department of General Internal Medicine, Nara City Hospital, Nara; b Department of General Internal Medicine, Nerima Hikarigaoka Hospital, Tokyo; c Department of Nephrology, Endocrinology, and Diabetes, Tokyo Bay Urayasu Ichikawa Medical Center, Chiba; d Department of General Internal Medicine, Thrombosis and Hemostasis Center, Saitama Medical University Hospital, Saitama, Japan.

**Keywords:** acquired factor V deficiency, coagulopathy, factor V inhibitor, mixing study

## Abstract

**Rationale:**

Acquired inhibitors of coagulation are antibodies that either inhibit the activity or increase the clearance of a clotting factor. Acquired factor V deficiency is a rare coagulation disorder, and it can sometimes be life threatening.

**Patient concerns:**

We describe a case of a 90-year-old Japanese male with acquired factor V deficiency. He was previously misdiagnosed with congenital factor V deficiency when he presented with hemoptysis and a negative factor V inhibitor test result at a different hospital 5 years earlier. Coagulopathy recurred with ecchymosis when he sustained a bruise after falling on a bush.

**Diagnosis:**

Although the factor V inhibitor test result was negative and a mixing study suggested a deficiency pattern, we diagnosed the patient with acquired factor V deficiency on the basis of no history of bleeding diathesis, a lack of response to multiple fresh frozen plasma transfusion, and clinical response to corticosteroid therapy.

**Interventions:**

Intravenous methylprednisolone was administered at 500 mg/day for 3 days, followed by oral prednisolone at 1 mg/kg/day.

**Outcomes:**

Coagulation test results improved and symptoms resolved 2 weeks after corticosteroid administration.

**Lessons:**

This case report suggests that clearance-facilitating antibodies exist without the presence of neutralizing inhibitors. When patients present with coagulation factor V deficiency in the absence of coagulation inhibitors, acquired factor V deficiency should also be considered.

## 1 Introduction

Acquired factor V deficiency is a rare hemostatic disorder caused by the presence of coagulation factor inhibitors, and it can sometimes be life threatening. The estimated incidence of acquired factor V deficiency is approximately 1/million individuals/year.^[1]^ In 2018, the Japanese Ministry of Health, Labor and Welfare designated autoimmune acquired factor V deficiency as an intractable disease. The most common acquired coagulation factor inhibitors are factor VIII inhibitors, which are mostly found in patients with acquired hemophilia. The incidence of acquired factor V deficiency caused by coagulation factor inhibitors is rare in Japan.^[2,3]^ Patient characteristics that are reportedly associated with the presence of factor V inhibitors are variable and include prior history of surgery, use of antibiotics, exposure to bovine protein, infections, malignancies, autoimmune diseases, and transfusion.^[3]^ Furthermore, approximately 20% of patients with acquired factor V inhibitors are idiopathic.^[3]^ In this report, we present the case of a patient with acquired factor V deficiency who was previously misdiagnosed with congenital factor V deficiency because of a negative factor V inhibitor test result. We diagnosed the patient with acquired factor V deficiency based on his past medical history, a lack of response to multiple units of fresh frozen plasma (FFP) transfusion, and successful treatment with corticosteroid therapy. Similar cases have previously been reported for acquired factor V deficiency without factor V inhibitor test result.^[4–6]^

## 2 Case report

**Figure 1:**
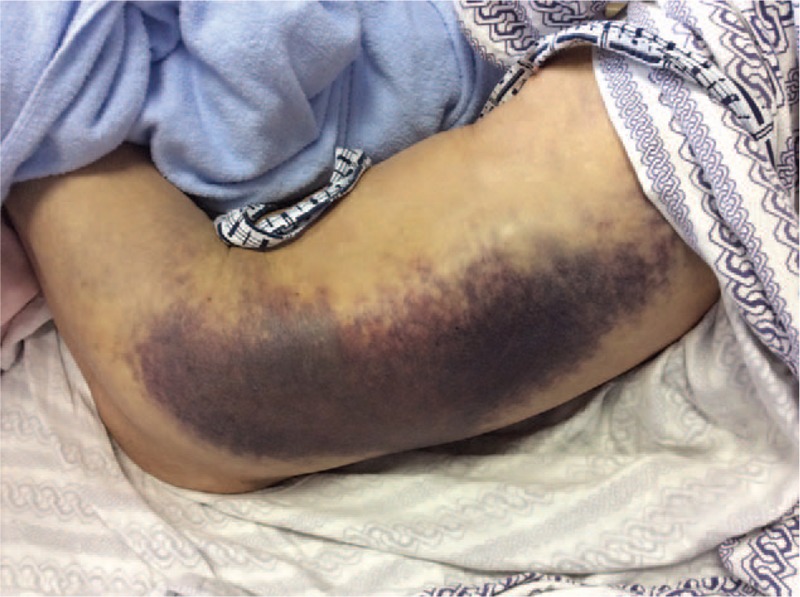
An abdominal examination revealed left flank firmness with large ecchymosis.

**Figure 2:**
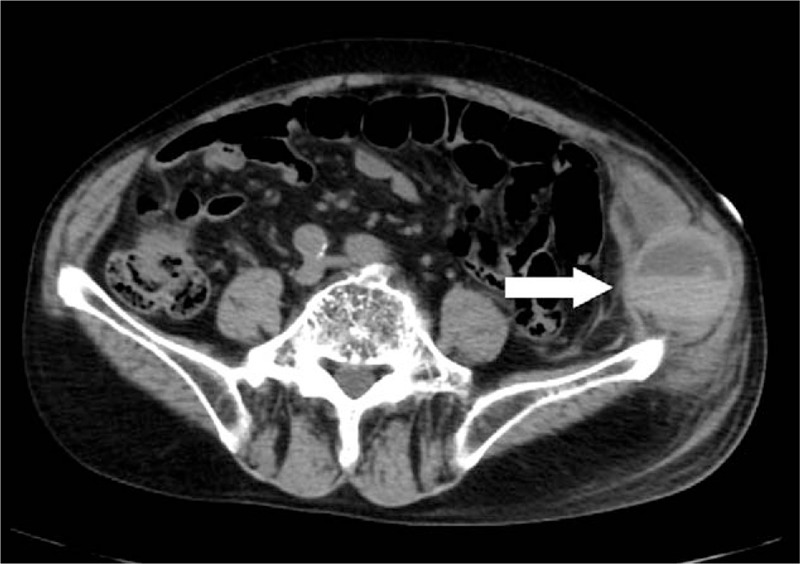
Computed tomography of the abdomen revealed left firmness and intramuscular hematoma (arrow head). Extravasation was not observed using a contrast agent.

**Figure 3:**
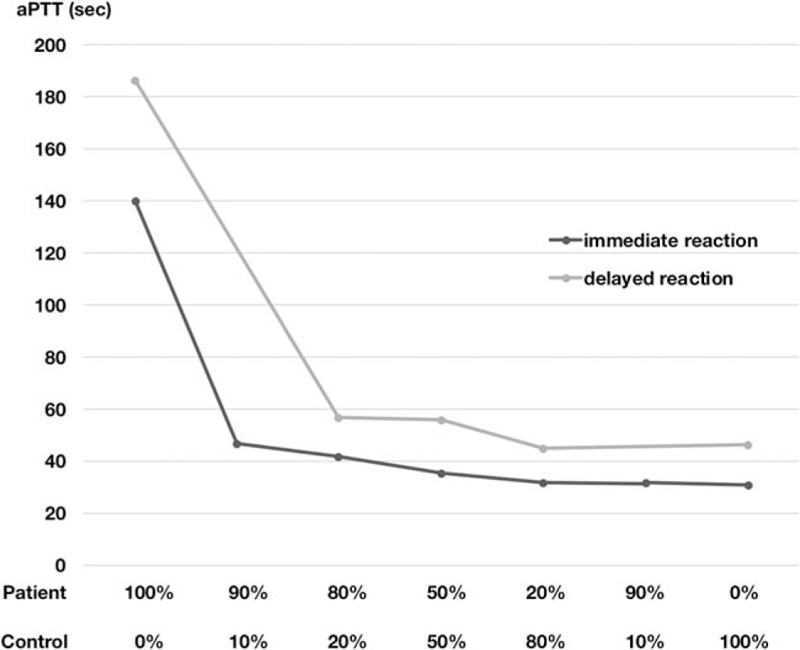
Mixing study. Plasma samples from the patient and a healthy volunteer were mixed at the indicated ratio. The aPTT was measured 2 hours after the incubation of samples at 37°C. aPTT was prolonged, and the curve was downward and convex, suggesting deficiency pattern. aPTT = thromboplastin time.

A 90-year-old Japanese male with a history of pulmonary tuberculosis presented with left flank pain and bruising after a fall that persisted for 5 days. He had no prior history of abnormal bleeding or recent surgery and no family history of bleeding diathesis. However, he had visited another hospital 5 years prior due to hemoptysis, where he was diagnosed with congenital factor V deficiency. His hemoptysis could be spontaneously healed without medication and transfusion. He denied having any other significant past medical history other than obsolete pulmonary tuberculosis. He was not taking any nutritional supplement or medication. A physical examination on admission revealed that he was afebrile and hemodynamically stable. He showed left flank firmness with a large ecchymosis (Fig. 1). Laboratory analysis revealed a hemoglobin level of 12.3 g/dL and platelet count of 296,000/μL. Complete blood count, electrolyte and creatinine levels, and liver function test results were normal. Prothrombin time (PT) was prolonged at 40.4 s (reference value, 10.0–13.0 s), and activated partial thromboplastin time (aPTT) was prolonged at 287.7 s (reference value, 23.0–36.0 s). Additional coagulation tests included those for fibrinogen (828 mg/dL; reference value, 200–400 μg/mL), fibrin/fibrinogen degradation products (6.9 μg/mL; reference value, <5 μg/mL), D-dimer (2.8 μg/mL; reference value, <5 μg/mL), and protein induced by vitamin K absence or antagonist II (PIVKA-II) (12 mAU/mL; reference value, 0–40 mAU/mL). The hepaplastin test and thrombotest were not used and test results for lupus anticoagulant and anticardiolipin antibodies were negative. The activities of factors II and X were 87% and 80%, respectively, whereas the activity of factor V was <1.0%. Moreover, test results for factor V inhibitors were negative in the Bethesda assay. Computed tomography of the abdomen revealed left intramuscular hematoma, and no extravasation was observed using a contrast agent (Fig. 2). A mixing study was conducted using plasma samples from the patient and a healthy volunteer. The aPTT curves were convex and downward, and both of the initial and 2 hour post-incubation at 37°C curves suggested a deficiency pattern (Fig. 3).

**Figure 4:**
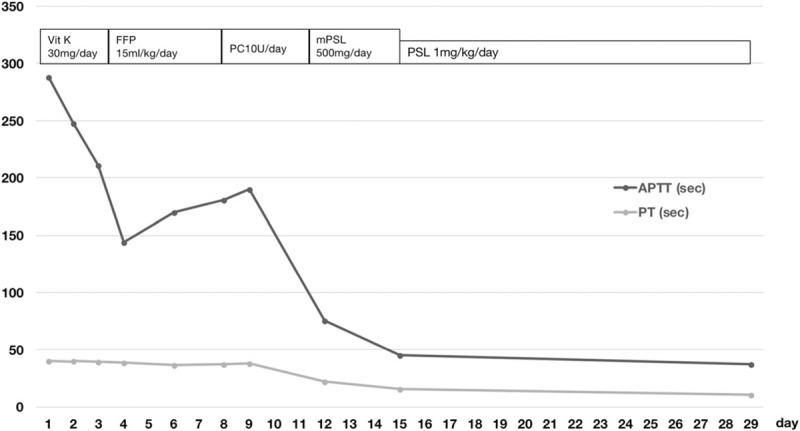
Clinical course of the patient with acquired factor V deficiency. aPTT = thromboplastin time, PT = prothrombin time.

Following hospitalization, the patient was administered intravenous vitamin K at 30 mg/day for the first 2 days because the result for PIVKA-II was uncertain. He was then administered FFP at 15 mL/kg/day for the next 5 days. However, the coagulation test value did not normalize. Subsequently, platelet concentrates were administered at 10 units/day for 3 days given the presence of coagulation factor V in platelets.^[7]^ Multiple units of transfusion were ineffective in treating his coagulopathy (Fig. 4); however, this lack of response to multiple transfusions is consistent with acquired coagulation deficiency but not with congenital coagulation deficiency. Although test results for factor V inhibitors were negative and a mixing study demonstrated a deficiency pattern, the patient was clinically diagnosed with acquired factor V deficiency. As a result of this, intravenous methylprednisolone was administered at 500 mg/day for 3 days, followed by oral prednisolone at 1 mg/kg/day. Coagulation test results improved and symptoms resolved 2 weeks after corticosteroid administration (Fig. 4). The patient was discharged from the hospital at day 15. He has been followed up as an outpatient and has shown no recurrence of coagulopathy for 1 year.

## 3 Discussion

Although the precise mechanisms underlying the development of coagulation inhibition remain unclear, previous studies on acquired factor V deficiency have suggested 3 distinct mechanisms, namely, spontaneous autoantibodies, antibodies cross-reacting with anti-bovine factor V, and alloantibodies.^[8]^ Factor V inhibitors are most often associated with surgical procedures in which topical bovine thrombin is used.^[4]^ In this case, the patient's medical history and clinical examination did not indicate the presence of an autoimmune disease or exposure to bovine thrombin.

In addition, it is known that the clearance-facilitating antibodies, and not neutralizing antibodies, to coagulation factors account for acquired factor V deficiency in certain cases.^[4,5]^ Test results for factor V inhibitors may be negative if a patient has clearance-facilitating antibodies. Various studies have reported that the detection of certain antibodies to factor V may require prolonged incubation periods of mixing studies and that this time dependence of factor V inhibitors appears to be most clinically significant at low titers.^[4]^ A similar, but more pronounced, effect has been observed with factor VIII inhibitors, which has led to the recommendation that mixing studies should be conducted using a 2-hour incubation period.^[9]^ Conversely, inhibitors of factors other than factors V and VIII do not appear to exhibit such time dependence.^[4]^ In this case, we could not indicate factor V inhibitors with a 2-hour incubation period of mixing studies. It is known that coagulation factor V is present in plasma (80%) and platelets (20%).^[7]^ Different antibodies to factor V differ in their ability to access factor V stored in platelets and to recognize the C2 domain of the light chain of factor V.^[10,11]^ At low titers, certain low-affinity or low-avidity antibodies may take longer to interact with factor V.^[12]^ Based on these considerations, there is a possibility that clearance-facilitating antibodies exist in the absence of neutralizing inhibitors. In this case, the absence of a family history of bleeding diathesis and a medical history of bleeding tendency, the ineffectiveness of FFP transfusion, and the clinical response to corticosteroids supported the diagnosis of acquired factor V deficiency. When patients present with coagulation factor V deficiency in the absence of coagulation inhibitors, acquired factor V deficiency should also be considered.

## 4 Conclusion

This study presents the case of a symptomatic Japanese patient diagnosed with acquired factor V deficiency in the absence of neutralizing factor V inhibitors. It is important to accurately assess the etiology of coagulation factor V deficiency using laboratory analysis involving a mixing study and whether it is congenital or acquired to be able to select an appropriate treatment.

## Author contributions

**Conceptualization:** Kiyoshi Takemoto, Osamu Hamada, Koichi Kitamura, Naoki Fujiwara, Yoshitaka Miyakawa.

**Supervision:** Yoshitaka Miyakawa.

**Validation:** Yoshitaka Miyakawa.

Kiyoshi Takemoto orcid: 0000-0001-8072-6298.

## References

[1] Collins P Macartney N Davies R A population based, unselected, consecutive cohort of patients with acquired haemophilia A. Br J Haematol 2004;124:86–90. 14675412 10.1046/j.1365-2141.2003.04731.x

[2] Kunimoto H Miyakawa Y Okamoto S Acquired factor V deficiency and mini literature review. Haemophilia 2012;18:e60–87. 21910794 10.1111/j.1365-2516.2011.02650.x

[3] Ang AL Kuperan P Ng CH Ng C HJ Acquired factor V inhibitor. A problem-based systematic review. Thromb Haemost 2009;101:852–9. 19404538

[4] Lipshitz J Chelliah T Alert L A case of factor V inhibitor with complete correction of the PT and aPTT upon mixing. Am J Hematol 2012;87:313–5. 22139582 10.1002/ajh.22245

[5] Sosa IR Ellery P Mast A Acquired factor V deficiency in a patient without evidence of a classical inhibitor. Haemophilia 2014;20:e81–3. 24118596 10.1111/hae.12280 PMC3870044

[6] Quek JKS Wong WH Tan CW Acquired factor V deficiency in a patient with myeloma and amyloidosis. Thromb Res 2018;164:1–3. 29427868 10.1016/j.thromres.2018.01.045

[7] Tracy PB Eide LL Bowie EJ Mann KG Radioimmunoassay of factor V in human plasma and platelets. Blood 1982;60:59–63. 7082847

[8] Ortel TL Clinical and laboratory manifestations of anti-factor V antibodies. J Lab Clin Med 1999;133:326–34. 10218762 10.1016/s0022-2143(99)90062-8

[9] Lossing TS Kasper CK Feinstein DI Detection of factor VIII inhibitors with the partial thromboplastin time. Blood 1977;49:793–7. 856360

[10] Feinstein DI Rapaport SI McGehee WG Patch MJ Factor V anticoagulants: clinical, biochemical, and immunological observations. J Clin Invest 1970;49:1578–88. 4194089 10.1172/JCI106375 PMC322637

[11] Ortel TL Quinn-Allen MA Keller FG Localization of functionally important epitopes within the second C-type domain of coagulation factor V using recombinant chimeras. J Biol Chem 1994;269:15898–905. 7515064

[12] Ortel TL Moore KD Quinn-Allen MA Inhibitory anti-factor V antibodies bind to the factor V C2 domain and are associated with hemorrhagic manifestations. Blood 1998;91:4188–96. 9596666

